# Impact of Moderate‐Intensity Multicomponent Training on Cardiometabolic Health‐Related Outcomes in Older Adults With Overweight and Obesity: A 9‐Month Quasi‐Experimental Single‐Arm Pretest‐Posttest Study

**DOI:** 10.1111/ajag.70176

**Published:** 2026-05-11

**Authors:** Enzo Berbery, Vitor de Salles Painelli, Paulo Vitor Suto Aizava, Marilene Ghiraldi de Souza Marques, Endriw Domingues Noronha, Déborah Cristina de Souza Marques, Kan Oishi, Jordan Hernandez‐Martinez, Pablo Valdés‐Badilla, Braulio Henrique Magnani Branco

**Affiliations:** ^1^ Interdisciplinary Laboratory for Intervention in Health Promotion Postgraduate Program in Health Promotion Cesumar Institute of Science, Technology and Innovation Maringa Parana Brazil; ^2^ Physical Education Department State University of Maringa Maringa Parana Brazil; ^3^ Postgraduation Program in Movement Science Federal University of Piauí Teresina Piauí Brazil; ^4^ Faculty of Education Saga University Saga Japan; ^5^ Department of Physical Activity Sciences Universidad de Los Lagos Osorno Chile; ^6^ Department of Physical Activity Sciences Faculty of Education Sciences, Universidad Católica del Maule Talca Chile; ^7^ Sports Coach Career Faculty of Life Sciences, Universidad Viña del Mar Viña del Mar Chile

**Keywords:** geriatric assessment, health promotion, physical exercise

## Abstract

**Objective:**

The aim of this study was to investigate the effects of a moderate‐intensity multicomponent training (MCT) program on body composition, physical function, glycaemic control and lipid profiles in overweight and obese older people.

**Methods:**

This single‐arm quasi‐experimental study included 48 male (*n* = 10) and female (*n* = 38) overweight (*n* = 27) and obese (*n* = 21) older individuals (69.2 ± 4.3 years; 76.4 ± 12.3 kg; 1.60 ± 0.1 m; 30.3 ± 4.7 kg·m^2^) who were subjected to a 36‐week moderate‐intensity MCT intervention, including strength, stretching, endurance and balance exercises, twice a week, along with nutritional counselling and psychoeducation. Body composition, physical function (upper‐ and lower‐body strength endurance, lower‐body flexibility and dynamic balance), fasting plasma glucose, total cholesterol (TC), low‐density lipoprotein (LDL‐c) and high‐density lipoprotein (HDL‐c) levels were assessed before and after 12, 24 and 36 weeks of intervention. Food consumption was evaluated at baseline and after 36 weeks.

**Results:**

Except for TC (*p* = 0.58; *W* = 0.014), a significant improvement was detected in LDL‐c (*p* < 0.001; *W* = 0.132) and HDL‐c (*p* < 0.001; *W* = 0.195), as well as in fasting glucose (*p* < 0.001; *W* = 0.556). Upper‐ and lower‐body strength endurance (both *p* < 0.0001, *W* = 0.447 and *W* = 0.175, respectively), lower‐body flexibility (*p* = 0.02; *W* = 0.068) and dynamic balance (*p* = 0.04; *W* = 0.060) also improved in response to MCT. However, all body composition outcomes remained unchanged throughout the MCT intervention (all *p* > 0.05). Similarly, no significant differences were identified in the food intake variables (all *p* > 0.05).

**Conclusions:**

Our moderate‐intensity MCT program effectively improved physical function, glycaemic control and lipid profile, but not body composition, in overweight and obese older adults.

## Abstract (highlights)

**Policy Impact:**

The study found that overweight and obese older people experienced significant improvements in muscle function and metabolic‐related outcomes following a moderate‐intensity multicomponent training program. These findings suggest that such training method may be a cost‐effective strategy for enhancing key health parameters in this population.

**Practice Impact:**

The study provides evidence that moderate‐intensity multicomponent training may help mitigate the decline in these key health parameters among overweight and obese older adults. These findings offer valuable support for health professionals when making therapeutic decisions for this population.

## 1 Introduction

More than 60% of Brazilian older adults (60 years and older) living in state capitals are overweight, and more than 20% live with both obesity and multiple chronic conditions [1]. This combination is linked to a threefold increase in the risk of disability, reduced quality of life and a greater likelihood of hospitalisation, nursing home placement and premature death [2, 3]. The public health impact is significant, contributing to rising healthcare costs and a heightened risk of developing frailty [4]. Exercise training is regarded as a first‐line low‐cost treatment for overweight and obese individuals, as it has been shown not only to reduce body fat but also to preserve lean mass—an especially critical goal in overweight and obese older adults to help prevent the onset of disability [5, 6]. Moreover, exercise training for this population is also implicated in the enhancement of noncommunicable disease‐related metabolic parameters, such as glycaemic control and lipid profile [7, 8], as well as in the improvement of physical function outcomes, such as muscle strength endurance, flexibility and balance, which may help prevent/counter frailty [9].

In this sense, multiple studies have sought to determine the therapeutic value of distinct exercise training modalities for overweight and obese older adults [5, 10]. A training method that has gained attention among the older population in the last 15 years is multicomponent training (MCT), which has been highlighted as a cost‐effective approach incorporating balance, strength, stretching and endurance exercises [11]. There is compelling evidence that MCTs may enhance muscle, cognitive and cardiorespiratory function in older adults [12], as well as biomarkers of lipid profile and glycaemic control [13]. Furthermore, MCT has been shown to be more efficient than other training methods for improving muscle strength [14], lipid profile [15] and health‐related quality of life within this age group [16, 17]. Despite the well‐established effects of MCTs on physical function and metabolic‐ and health‐related parameters in older people, the specific effects of MCT on body composition remain inadequately characterised. Although systematic reviews have examined MCT's effects on functional fitness and frailty in older adults, they have identified considerable heterogeneity and inconsistent findings regarding body composition outcomes. Specifically, some studies demonstrate beneficial effects on body fat and lean mass [18, 19], whereas others report no significant changes [14, 20, 21], underscoring the need for additional well‐designed investigations. Studies specifically targeting overweight and obese older adults are particularly scarce [22, 23], and these investigations display substantial heterogeneity in methodological aspects, including sample size, intervention duration, training intensity and exercise volume. This variability across studies has cast ambiguity in literature, limiting the generalisability of findings. Moreover, although contemporary evidence demonstrates that frail older adults can safely engage in and benefit from various MCT intensities, including moderate‐intensity programs as well as higher‐intensity protocols incorporating power‐oriented resistance training and high‐intensity interval training (HIIT) [24], the majority of published MCT interventions for overweight and obese older adults have employed low‐ to moderate‐intensity protocols. Consequently, it remains unclear whether the inconsistent body composition findings reflect insufficient training stimulus (in terms of intensity and/or volume) or whether body composition adaptations to MCT are inherently modest regardless of prescription parameters in this population.

Given the strong therapeutic potential of MCT for overweight and obese older individuals, along with the equivocality of the existing evidence, there is a clear need for further research in this area. The aim of this study, therefore, was to investigate the effects of 36‐week moderate‐intensity MCT on body composition (i.e., lean mass, free fat mass, fat mass and body fat percentage) in a significant sample of overweight and obese older adults. To further assess the efficacy of MCT, we also examined glycaemic control (i.e., fasting glucose), lipid profile‐related parameters (i.e., total cholesterol, low‐density lipoprotein cholesterol [LDL‐c] and high‐density lipoprotein cholesterol [HDL‐c]) and physical function (i.e., strength endurance, flexibility and balance). It was hypothesised that MCTs would improve body composition, physical function, glycaemic control and lipid profile in our sample of participants.

## 2 Methods

### 2.1 Participants and Experimental Design

Using gait speed as the dependent variable and MCT as the treatment, we calculated that 26 individuals were required for this study, assuming a moderate correlation among repeated measures of 0.5, a power of 0.90, an effect size of 0.27 [25] and an alpha error of 0.05 (G*Power). For this quasi‐experimental, repeated measures study, 86 participants were recruited via multiple communication channels (e.g., television, radio and social media). To be considered for the study, participants had to fulfil the following criteria: (a) being able to understand and sign the informed consent; (b) being 60 years or older; (c) being overweight (body mass index [BMI] ≥ 25.0 kg·m^2^) or obese (BMI ≥ 30.0 kg·m^2^); (d) having a fat mass ≥ 21.0 kg [26]; and (e) having medical approval to engage in exercise training. Participants using medications to manage certain medical conditions (e.g., hypertension) were included if their treatment regimen had remained stable for at least 6 months prior to study engagement and did not change throughout the study. Participants diagnosed with debilitating neurological disorders (e.g., Alzheimer's disease or Parkinson's disease), cancer or cardiac arrhythmias were excluded. Twenty‐one individuals either declined participation or did not meet the eligibility criteria. As a result, 27 overweight and 21 obese male (*n* = 10) and female (*n* = 38) older participants (69.2 ± 4.3 years; 76.4 ± 12.3 kg; 1.60 ± 0.1 m; 30.3 ± 4.7 kg·m^2^) were enrolled in the study. The study protocol was registered under doi 10.17605/OSF.IO/3UXP9, and approved by the Committee of Ethics in Research of the University Center of Maringá (UniCesumar)—approval number: 3.373.307, with each participant providing written informed consent before enrolment in the study.

**FIGURE 1:**
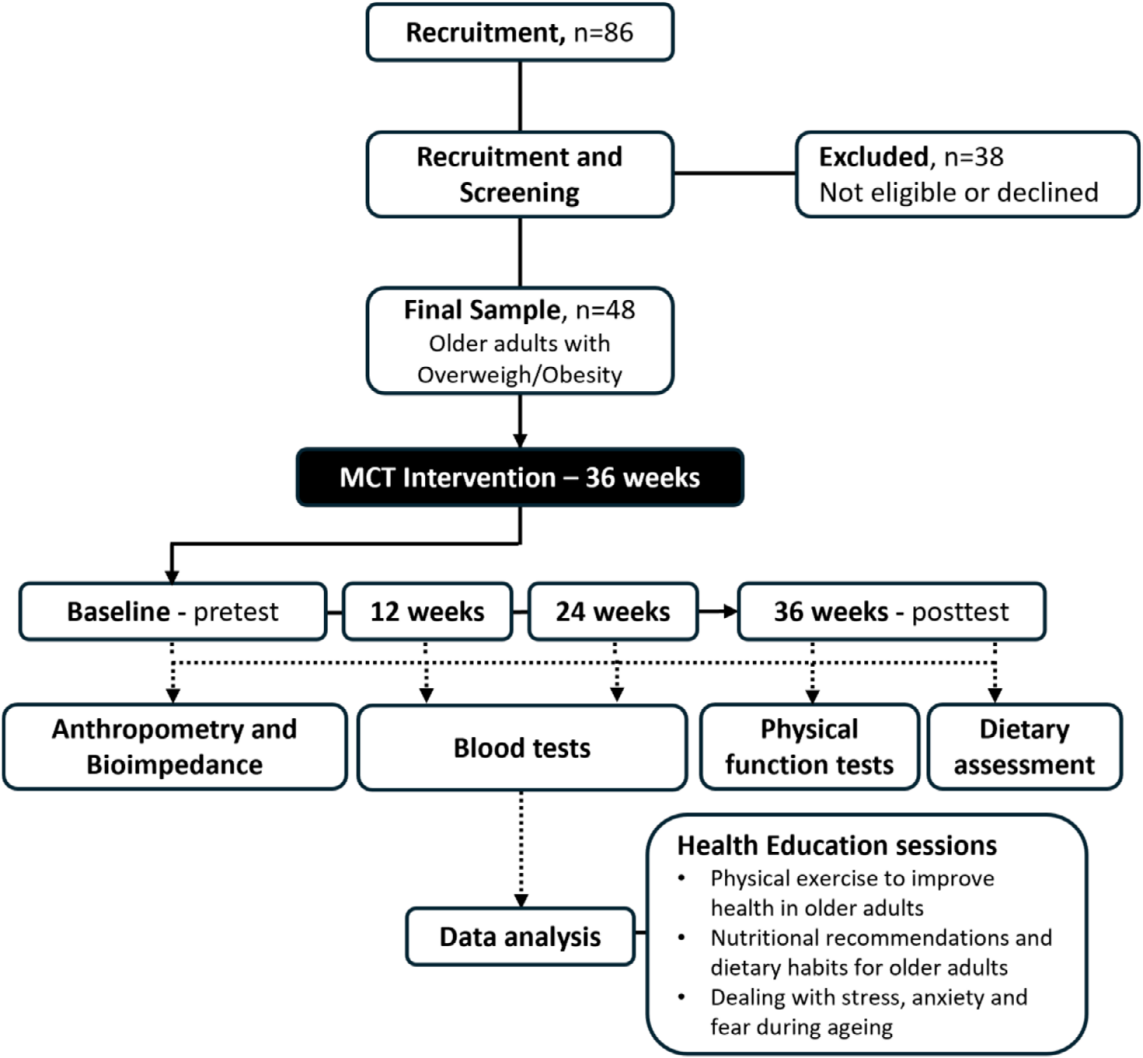
Experimental design.

Each participant completed a 36‐week MCT intervention. They were assessed at four time points: pre‐intervention (baseline), after 12 weeks, after 24 weeks and after 36 weeks of intervention, as illustrated in Figure 1. At each time point, assessments were conducted in the following order: (i) anthropometric measurement; (ii) bioimpedance analysis (BIA); (iii) blood sample collection; (iv) physical function assessment; and (v) dietary assessment. The participants were advised to abstain from unaccustomed exercise, caffeine and alcohol in the 24‐h prior to the assessments. Ad libitum water consumption was allowed during the physical function assessment. To improve adherence to the training protocol and reduce possible drop‐off caused by multiple evaluation periods, the participants received group discussions related to healthy habits. The topics of these talks were as follows: ‘Nutritional recommendations and dietary habits for older people’, ‘Physical exercise to improve health in older people’ and ‘Dealing with stress, anxiety and fear during ageing’. These discussions were delivered once a week by certified nutritionists and psychotherapists before the MCT sessions. All MCT sessions were carried out from March to November 2023 at the Cesumar Institute of Science, Technology and Innovation, and were supervised by exercise training professionals.

### 2.2 Body Composition and Anthropometric Assessment

Body composition was assessed via a tetrapolar BIA (InBody 570; Biospace Co. Ltd., Seoul, South Korea), with a 250 kg maximum capacity and 50 g margin of error. The participants removed their shoes and heavy clothing prior to BIA. All measurements were conducted at the same time of day under consistent conditions, with participants having an empty bladder to ensure uniformity across evaluations. The following variables were assessed: body weight (kg), body fat percentage (%), fat mass (kg), lean mass (kg) and fat‐free mass (kg). Previous studies have demonstrated that this BIA model has a correlation of *r* = 0.935 and *r* = 0.969 for fat mass and lean mass, respectively, with dual energy X‐ray absorptiometry (DXA) in older individuals [27].

Participants' height was measured with participants standing in full extension using a wall‐mounted stadiometer (Welmy R‐110; Santa Bárbara D'Oeste, Brazil) with a maximum capacity of 2.2 m and 0.1 cm margin error. A square was firmly positioned on the vertex of the head, compressing the hair as much as possible. The participants were instructed to take a deep breath and hold it before exhaling to ensure that accurate measurements were obtained. Body mass index was determined with the formula weight [kg]/height [m^2^], following World Health Organization guidelines [28].

### 2.3 Glycaemic Control and Lipid Profile Assessment

All participants fasted overnight prior to blood collection, which was performed via venipuncture in the antecubital fossa (cephalic, basilic or median cubital veins) via a 21‐gauge butterfly needle (BD Vacutainer Safety‐Lok, BD Biosciences, Australia). Four 4 mL Vacutainer tubes (BD Biosciences, Australia) were used—one containing citrate and three containing ethylenediaminetetraacetic acid (EDTA). Blood samples for biochemical analysis were collected from all participants between 6:30 and 9:30 AM, centrifuged for 10 min at 3500 rpm and left to clot at room temperature. Serum analyses were conducted within 24 h of collection via a semiautomated biochemical and turbidimetric analyser (Urit‐8021A; MHLab, São Paulo, Brazil) using specific kits (Gold Analisa Diagnostica Ltda., Minas Gerais, Brazil). All analyses were conducted in triplicate by the same researcher. The outcomes included fasting plasma glucose, total cholesterol, HDL‐c and LDL‐c levels.

### 2.4 Physical Function Assessment

Physical function was assessed through the following tests: (i) back scratch test; (ii) sit and reach test; (iii) 30‐s chair stand test; (iv) arm curl test; (v) timed up‐and‐go test (TUG); and (vi) 6‐min walk test (6MWT). Each test was separated by a 2‐min interval.

Briefly, upper‐body flexibility was assessed via the back scratch test [29], whereas lower‐body flexibility was evaluated via the sit and reach test [29]. The back scratch test was performed in a standing position, where participants reached one hand over their shoulder and down their back, with the other hand reaching up from behind the waist toward the first. They then switched arms and repeated the movement. The distance between the tips of the middle fingers was measured as follows: a score of 0 was given if the fingers just touched; a negative value (in centimetres) was given if there was a gap; and a positive value was given if the fingers overlapped. In the sit and reach test, participants sat on a chair placed against a wall for stability and extended one leg forward at a time, keeping the other foot flat on the floor. They reached toward the toes of the extended leg. A score of zero was given if the fingers just touched the toes; a negative value (in centimetres) was given if they fell short; and a positive value was given if the fingers extended beyond the toes.

In the 30‐s chair stand test, participants began the test seated, with their arms crossed at the chest level. From this position, they were instructed to stand up and sit down as many times as possible within 30 s [30]. The total number of repetitions was registered as the lower‐body strength endurance. Similarly, upper‐body strength endurance was evaluated via the 30‐s arm curl test, where participants had to perform as many bicep curls as possible for 30 s. An 8 lb. dumbbell was employed for men, and a 5 lb. dumbbell was employed for women, whereas the total number of repetitions was recorded [29].

Dynamic balance/gait performance was assessed through the TUG test, in which participants stood up from a chair, walked three metres at their usual pace, turned around, walked back to the chair and sat down [31]. The total time to complete the task was recorded. Finally, the 6MWT was used to assess walking endurance. The participants were instructed to walk as quickly and safely as possible for 6 min, aiming to cover the greatest distance possible along a 30‐m walkway with turns at each end. The total distance walked during the 6MWT was recorded [32].

### 2.5 Dietary Assessment

The participants' food intake was assessed pre‐ and post‐MCT via three 24‐h food diaries taken on separate days (2 weekdays and 1 weekend day). Energy and macronutrient intake were subsequently analysed by a certified nutritionist via specific software (Dietbox, Rio Grande do Sul, Brazil). Food intake was recorded at baseline and after 36 weeks of intervention.

### 2.6 Multicomponent Training Intervention

The MCT program was started approximately one week after the pre‐intervention assessments. It focused on endurance, strength, stretching and balance exercises to improve physical function. Each week, the participants completed two 60‐min MCT sessions, which were supervised by two specialised exercise training professionals. Each session had a maximum of 16 participants and an instructor‐to‐participant ratio of 1:8. Instructors always trained in the same group of participants and at the same time of the day to reduce interprofessional variability and circadian variation‐derived influence [33].

Each session consisted of 10‐min of balance exercises, followed by 20‐min of strength training and 20‐min of endurance exercises. A cool down was applied in the last 10‐min involving static stretching exercises. The balance training component comprises four tasks (walking forward/reverse/lateral, static balancing on one leg, unstable sitting balance and walking in a zigzag while bouncing a ball), which are performed for two sets of 30 s each. The strength training component included six exercises (knee flexion, leg press, knee extension, seated row, seated chest press) performed on a hoist machine. Initially, two sets of these exercises (12–15 repetitions each) were performed, and a 45‐s rest interval was allowed between the sets; gradually, this interval was changed to three sets (10–12 repetitions each), with a 60‐s rest interval between the sets being allowed. The intensity was monitored and adjusted through the OMNI scale [34], in which participants reported between 5 and 7 (‘somewhat hard’). The endurance exercise component included walking on a treadmill or stationary cycling. Intensity was monitored and adjusted through the Borg's rate of perceived exertion scale [35], in which participants should report between 12 and 14 (‘somewhat hard’). Finally, two sets of five static stretching exercises targeting the major strength trained muscles (hamstrings, quadriceps, gastrocnemius, deltoids and pectorals) were unilaterally performed. The participants were instructed to hold each position for 30 s until slight discomfort occurred [36]. During the MCT program, blood collection, body composition analyses and physical function assessments were repeated at 12‐week intervals, as described in the ‘Participants and Experimental Design’ section.

### 2.7 Statistical Analysis

Data were presented as the mean (M) ± standard deviation (SD) and 95% confidence interval (95% CI), whereas relative change (Δ%) values were calculated by: [(Post‐Pre)/(Pre)*100]. Normality was verified through the *Shapiro–Wilk* test; as none of the variables met the normality assumption, comparisons across time points were performed using Friedman test for repeated measures. Food intake data were compared via the Wilcoxon test. To calculate effect size, *Kendall's W* was used [37] where 0 indicates no association and 1 indicates a perfect relationship among measures. Statistical analyses were performed by the SPSS statistical software (27.0 version), with significance set at *p* < 0.05.

## 3 Results

### 3.1 Anthropometric and Body Composition Outcomes

No significant main effect of time was detected for body weight, BMI, body fat percentage, fat‐free mass, lean mass or fat mass (all *p* > 0.05), indicating that they remained unchanged across the 36 weeks of MCT (Table 1).

**TABLE 1:** Anthropometric and body composition outcomes at baseline and after 12, 24 and 36 weeks of intervention.

Body composition	Time measurements					*p*	*W*	Δ%
		Baseline	12 weeks	24 weeks	36 weeks			
Body weight (kg)	M ± SD	74.9 ± 14.3	75.3 ± 14.5	74.5 ± 14.6	74.2 ± 7.7	0.46	0.018	−0.5
	95% CI	70.7 to 79.0	71.1 to 79.5	70.3 to 78.9	70.1 to 78.4			
BMI (kg·m^2^)	M ± SD	30.2 ± 4.7	29.3 ± 5.4	29.4 ± 5.3	29.5 ± 5.3	0.11	0.041	−2.3
	95% CI	28.9 to 31.6	27.7 to 30.9	27.8 to 30.9	27.9 to 31.0			
Body fat (%)	M ± SD	39.0 ± 8.9	38.9 ± 9.0	39.0 ± 8.7	39.4 ± 8.3	0.70	0.010	1.9
	95% CI	36.5 to 41.6	36.2 to 41.5	36.4 to 41.5	37.0 to 41.8			
Fat‐free mass (kg)	M ± SD	44.9 ± 8.2	45.0 ± 8.5	45.9 ± 8.8	45.3 ± 8.6	0.99	0.001	1.0
	95% CI	42.5 to 47.8	42.6 to 47.5	43.3 to 48.4	42.8 to 47.8			
Lean mass (kg)	M ± SD	42.4 ± 7.7	42.4 ± 8.0	42.5 ± 8.0	42.7 ± 7.9	0.84	0.006	0.8
	95% CI	40.1 to 44.6	40.1 to 44.7	40.2 to 44.9	40.4 to 45.0			
Fat mass (kg)	M ± SD	29.6 ± 11.1	29.8 ± 10.9	29.6 ± 10.7	30.0 ± 10.4	0.90	0.004	3.3
	95% CI	26.4 to 32.9	26.6 to 32.9	26.5 to 32.7	27.0 to 33.0			

*Note:* *n* = 48; No significant difference was observed. Abbreviations: 95% CI, 95% confidence interval; BMI, body mass index; M, mean; SD, standard deviation; *W*, Kendall's *W*; Δ%, delta variation.

### 3.2 Blood Biochemical Outcomes

**FIGURE 2:**
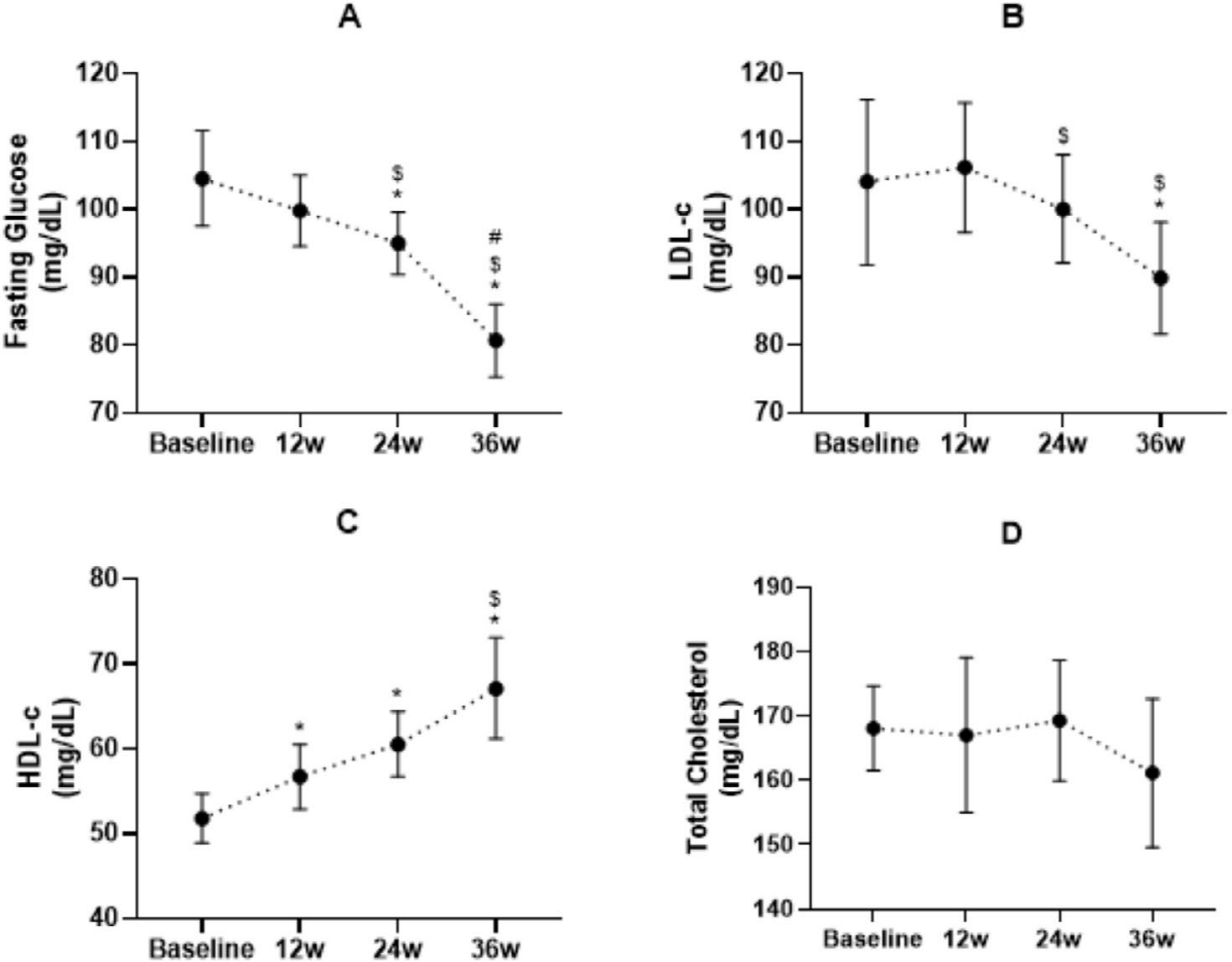
Blood biochemical outcomes at baseline and after 12, 24 and 36 weeks of intervention. *N* = 48. Data were expressed in Mean and 95% Confidence Interval; Panel A = Fasting glucose; Panel B = LDL‐c—Low‐density lipoprotein; Panel C = HDL‐c—High‐density lipoprotein; Panel D = Total cholesterol; 12w = 12 weeks; 24w = 24 weeks; 36w = 36 weeks. * Significant difference observed from baseline moment (*p* < 0.05). ^$^Significant difference observed from 12 weeks (*p* < 0.05). ^#^Significant difference observed from 24 weeks (*p* < 0.05).

Except for total cholesterol (Figure 2, panel D; *p* = 0.58; *W* = 0.014; Δ% = −3.6), a significant difference was detected for fasting glucose (Figure 2, panel A; *p* < 0.001; *W* = 0.556; Δ% = −20.1), LDL‐c (Figure 2, panel B; *p* < 0.001; *W* = 0.132; Δ% = −1.6) and HDL‐c (Figure 2, panel C; *p* < 0 0.001; *W* = 0.195; Δ% = 33.1), meaning that they all changed compared with baseline values.

### 3.3 Physical Function Outcomes

No significant main effect of time was shown for the total distance achieved in the back scratch test (*p* = 0.25; Table 2) or in the 6MWT (*p* = 0.21; Table 2). On the contrary, a significant main effect of time was demonstrated for total distance achieved in the sit and reach test (*p* = 0.02; Table 2), as well as for the total number of repetitions achieved in the 30‐s chair stand test and in the arm curl test (both *p* < 0.001; Table 2). Similarly, a significant main effect of time was detected in the time to complete the TUG test (*p* = 0.04; Table 2).

**TABLE 2:** Physical function outcomes at baseline and after 12, 24 and 36 weeks of intervention.

Physical function tests	Time measurements					*p*	*W*	Δ%
		Baseline	12 weeks	24 weeks	36 weeks			
Back scratch (cm)	M ± SD	−6.6 ± 14.9	−7.1 ± 15.0	−7.0 ± 14.2	−7.8 ± 14.6	0.25	0.029	8.1
	95% CI	−10.9 to 2.3	−11.5 to 2.8	−11.1 to 2.9	−12.0 to 3.6			
Sit and reach (cm)	M ± SD	0.7 ± 7.6	1.6 ± 11.6^a^	0.3 ± 11.2	1.8 ± 10.1^a^	0.02	0.068	60.6
	95% CI	−1.5 to 2.9	−1.8 to 4.9	−3.0 to 3.5	−1.1 to 4.8			
30‐s CST (rep)	M ± SD	14 ± 5	15 ± 4	14 ± 5	17 ± 5^a^, ^b^	< 0.001	0.175	17.7
	95% CI	12.9 to 15.8	14.0 to 16.5	13.0 to 15.7	15.2 to 18.2			
Arm curl (rep)	M ± SD	15 ± 5	20 ± 8^a^	19 ± 8^a^	21 ± 8^a^, ^b^	< 0.001	0.447	57.6
	95% CI	13.4 to 16	17.5 to 22.1	16.8 to 21.6	18.9 to 23.4			
TUG (s)	M ± SD	7.7 ± 2.2	7.3 ± 2.1	7.3 ± 1.8	7.1 ± 1.4^a^	0.04	0.060	−5.6
	95% CI	7.1 to 8.3	6.7 to 7.9	6.8 to 7.8	6.7 to 7.5			
6MWT (m)	M ± SD	494.8 ± 92.1	509.3 ± 95.7	510.4 ± 95.6	513.9 ± 71.6	0.18	0.034	5.9
	95% CI	468.1 to 521.6	481.5 to 537.1	482.6 to 538.1	493.1 to 534.7			

*Note:* *n* = 48. Abbreviations: 30‐s CST, 30‐s chair stand test; 6MWT, 6‐min walk test; 95% CI, 95% confidence interval; M, mean; SD, standard deviation; TUG, timed up and go; *W*, Kendall's *W*; Δ%, delta variation.

^a^ Significantly different observed from baseline moment (*p* < 0.05).

^b^ Significantly different observed from 24 weeks (*p* < 0.05).

### 3.4 Food Intake

No significant differences in food intake were detected in energy, carbohydrate, fat or protein intake between pre‐MCT and post‐MCT (all comparisons *p* > 0.05; Table 3).

**TABLE 3:** Energy and macronutrient intake at baseline and after 36 weeks of intervention.

Energy and macronutrient	Measurement times			*p*	Δ%
		Baseline	36 weeks		
Energy (kcal)	M ± SD	1523.4 ± 481.2	1525.1 ± 588.7	0.81	5.4
	95% CI	1375.3 to 1671.5	1343.9 to 1706.3		
Protein (g)	M ± SD	65.1 ± 21.5	70.1 ± 37.5	0.65	16.1
	95% CI	58.5 to 71.7	58.5 to 81.6		
CHO (g)	M ± SD	207.5 ± 85.4	184.3 ± 66.2	0.54	3.9
	95% CI	178.2 to 236.9	164.0 to 204.7		
Fat (g)	M ± SD	46.5 ± 17.3	56.4 ± 34.7	0.25	46.1
	95% CI	41.2 to 51.9	45.7 to 67.0		
Protein (g/kg/day)	M ± SD	1.0 ± 0.6	1.0 ± 0.5	0.84	−0.2
	95% CI	0.8 to 1.2	0.8 to 1.2		

*Note:* n/total = 43; No significant difference was observed. Abbreviations: 95% CI, 95% confidence interval; CHO, carbohydrate; M, mean; SD, standard deviation; Δ%, delta variation.

## 4 Discussion

The increasing prevalence of overweight and obesity among older individuals poses a significant public health challenge and complicates care for healthcare professionals. Obesity is linked to various metabolic disorders [38] and accelerates age‐related decline in physical function, increasing the risk of frailty and loss of independence in this population [39]. Our study demonstrated that a moderate‐intensity MCT program can improve physical function, glycaemic control and lipid profile in overweight and obese older adults. However, no changes in the anthropometric or body composition parameters were observed.

The present findings align with and extend recent systematic evidence examining various exercise modalities in populations with metabolic disorders. Al‐Mhanna et al. [40] demonstrated that combined aerobic and resistance training (CART) significantly improved glycaemic control, blood pressure, inflammation markers and cardiorespiratory fitness in patients with type 2 diabetes and overweight/obesity, suggesting that structured multimodal exercise approaches offer comprehensive cardiometabolic benefits even without significant weight reduction. Similarly, their subsequent meta‐analysis [41] confirmed that CART induced favourable changes in body composition, lipid metabolism and physical function in this population, reinforcing the therapeutic potential of combined training modalities. Recent investigations into high‐intensity exercise paradigms further illuminate the spectrum of effective interventions. Al‐Mhanna et al. [42] reported that HIIT improved multiple cardiometabolic markers in patients with diabesity, notably achieving metabolic benefits without substantial body composition changes, a pattern consistent with our moderate‐intensity MCT findings. The comparative analysis by Al‐Mhanna et al. [43] revealed that HIIT demonstrated superior insulin sensitivity improvements compared with moderate‐intensity continuous training, though both modalities yielded clinically meaningful glycaemic improvements. Furthermore, aerobic exercise alone has been shown to induce beneficial cardiometabolic adaptations in diabesity patients [44], suggesting that various exercise intensities and modes can effectively modulate metabolic health parameters in populations with concurrent metabolic disorders. Collectively, these findings underscore that multicomponent and combined exercise approaches, whether moderate‐intensity MCT as employed in the present study or higher‐intensity paradigms, consistently improve glycaemic control, lipid profiles and functional capacity across diverse populations with cardiometabolic impairments. The observation that metabolic benefits often occur independent of substantial body composition changes suggests that exercise exerts direct effects on insulin sensitivity, glucose metabolism, lipid handling and muscular adaptation that transcend simple energy balance considerations. This body of evidence supports the implementation of varied exercise modalities tailored to individual capabilities and preferences, with moderate‐intensity MCT representing a particularly feasible and safe option for older adults with overweight and obesity.

The current MCT program, which was aligned with the American College of Sports Medicine guidelines and incorporated strength, stretching balance and endurance exercises, resulted in significant improvements in both upper‐body and lower‐body strength endurance, lower‐body flexibility and dynamic balance [45]. Notably, strength endurance gains were achieved despite previous evidence indicating that strength training–induced enhancements in muscle strength are often diminished in obese individuals compared with their lean counterparts [46]. Additionally, combining strength and endurance training supposedly inhibits strength gains compared with strength training alone [47] and may also compromise endurance adaptations relative to endurance training alone [48]. Although the interference phenomenon of concurrent training has been classically proposed as a potential explanation for attenuated endurance or strength adaptations, evidence indicates that such effects are highly dependent on training characteristics, particularly high endurance volume and/or intensity [48]. In the present study, the endurance component was performed at moderate intensity and volume, conditions under which interference effects are less likely to occur. Moreover, systematic reviews and meta‐analyses suggest that interference is inconsistently observed and appears in a minority of concurrent training studies, especially in older adults, in whom combined training often elicits complementary rather than antagonistic adaptations [49]. Hence, although interference cannot be entirely ruled out, the lack of difference in 6MWT might be attributable to the relatively low endurance stimulus. Alternatively, both walking on a treadmill and stationary bicycles were allowed as endurance exercises, and more bicycles than treadmills were available in our training facility. Therefore, the unspecific nature of the endurance exercise employed in most exercise sessions during our MCT program may provide an alternative explanation for the lack of change in the total distance achieved in the 6MWT. In parallel, the MCT‐induced improvements in strength endurance observed in this study were accompanied by enhanced flexibility and dynamic balance in key functional tasks linked to frailty in overweight and obese older people [50], such as rising from a chair and walking speed. These abilities are essential for independent mobility and are strong predictors of one's capacity to carry out instrumental activities of daily living, including cooking, traveling and shopping. They are also known predictors of disability and the likelihood of nursing home admission [38, 39]. Therefore, these findings support the crucial role of MCT in enhancing overall physical function, underscoring its importance in managing frailty and slowing functional decline in older adults who are overweight or obese.

The distinct effects of exercise on some of the lipid profile outcomes detected in this study are anticipated, as previous examinations have shown that exercise does not have a uniform effect on all plasma lipids. In this sense, findings from comprehensive reviews [51, 52] indicate that exercise generally has minimal influence on total cholesterol and LDL‐c. The most consistent lipid‐related effects of exercise training are typically increases in HDL‐c [53, 54]. Despite the unexpected decrease in LDL‐c, our results are in line with these observations, as our MCT led to a significant increase in HDL‐c levels. These changes are consistent with findings from other studies employing MCT programs for older people [15, 55], as well as for individuals with coronary artery disease [56]. The mechanisms underpinning such changes are beyond the scope of four studies but might include increased skeletal muscle lipoprotein lipase activity and content, along with increased capillary density and a greater capacity for fatty acid uptake and utilisation [57]. At the same time, a significant decrease in fasting glucose was shown in response to our intervention, highlighting the clear clinical relevance of MCT for older adults with glucose intolerance or diabetes. Notably, the effect of MCT on fasting glucose, however, is not clear, and studies have failed to observe a positive effect [56, 57]. The absence of a change in fasting glucose in previous studies might reflect a potential ‘ceiling effect’ [58], in which participants from earlier investigations already had well‐controlled diabetes. Another possible explanation for the lack of improvement in fasting glucose in previous examinations could be the limited increase in lean mass resulting from the MCT intervention, as there seems to be a significant inverse relationship between gains in lean mass and reductions in fasting glucose levels [59]. Curiously, improved fasting glucose levels were identified in the current study without concomitant changes in fat‐free mass. Potentially unmonitored mechanisms underlying this improvement might include increased insulin sensitivity and increased GLUT4 expression and activity in skeletal muscle [60].

All body composition measures remained intact throughout the 36‐week MCT intervention. Although this finding contradicts our initial hypothesis, it is not unexpected, as most studies demonstrating improvements in body composition among older adults typically involve a combination of exercise training and calorie restriction [61, 62]. The nutritional counselling in our study was aimed exclusively at encouraging healthier eating habits rather than promoting weight loss. This was confirmed by the fact that the food intake data remained unchanged throughout the intervention. Nevertheless, previous investigations also did not report enhanced body composition in response to MCT interventions. For example, in a woman‐only sample, Rodrigues et al. [14] did not observe a significant decrease in body fat percentage or BMI after a 6‐month moderate‐intensity MCT intervention. In a sample with similar characteristics, Forte et al. [21] failed to demonstrate significant changes in body fat percentage, total body fat or total lean mass in response to an 8‐month moderate‐intensity MCT. Notably, some physical function outcomes worsened after the MCT program [21]. Using the DXA method, Leite et al. [20] also did not observe significant differences in BMI, body fat percentage or lean mass, although their moderate‐intensity MCT intervention was employed for only 12 weeks in a small sample of 21 older adults. In a larger sample of participants who were followed for a longer period than those in earlier studies, our findings agreed with the aforementioned results, suggesting that moderate‐intensity MCT has a negligible effect on the body composition of older adults, even those with overweight and obesity. Nevertheless, it should be emphasised that the moderate‐intensity, low‐volume MCT program in the present investigation was employed to cope with guidelines advising exercise interventions targeting people with frailty. It is still unclear whether high‐intensity or high‐volume MCT interventions are both effective in improving body composition and safe to implement.

This study has several limitations. Primarily, the sample of participants was selected by convenience, and the single‐arm quasi‐experimental design lacked randomisation. Likewise, although our 36‐week investigation might be considered one of the most extensive MCT interventions, offering a reasonable timeframe to evaluate exercise‐related effects, it is still shorter than the duration typically needed for overweight and obese older individuals to maintain long‐term engagement and achieve optimal improvements in quality of life. Finally, there are more accurate, reliable and sensitive methods than BIA for assessing body composition in response to exercise training, such as computed tomography and magnetic resonance imaging [63]. Future studies should attempt to employ such methods to assess body composition in response to MCT programs in overweight and obese older adults.

## 5 Conclusions

In summary, the current results provide evidence that moderate‐intensity MCT programs have beneficial effects on physical function, glycaemic control and lipid profile, but not on body composition in overweight and obese older adults; therefore, these programs should be considered in the care of this population.

## Funding

The Article Processing Charge for the publication of this research was funded by the Coordenação de Aperfeiçoamento de Pessoal de Nível Superior ‐ Brasil (CAPES) (ROR identifier: 00x0ma614). The authors declare that this study was supported by the Cesumar University (UniCesumar); Cesumar Institute of Science, Technology and Innovation (ICETI); Araucaria Foundation for Support of Scientific and Technology and Higher Education of Paraná via the University Without Borders Notice.

## Ethics Statement

The study was approved by the Committee of Ethics in Research of the University Center of Maringá (UniCesumar)—approval number: 3.373.307.

## Conflicts of Interest

The authors declare no conflicts of interest.

## Data Availability

The data that support the findings of this study are available from the corresponding author upon reasonable request.
